# Why We Need a Single Definition of Disruptive Behavior

**DOI:** 10.7759/cureus.2339

**Published:** 2018-03-18

**Authors:** Michelle A Petrovic, Adam T Scholl

**Affiliations:** 1 Anesthesiology, University of Central Florida; 2 Independent Researcher

**Keywords:** disruptive behavior, lateral violence, incivility, bullying, horizontal violence, nurse verbal abuse, behaviors that undermine a culture of safety, medical student mistreatment, disruptive clinicians, disruptive physicians

## Abstract

Disruptive behavior is known to produce a wide range of negative effects in healthcare, such as impacting patient safety, lowering employee morale, and decreasing employee retention. Healthcare organizations have worked towards eliminating disruptive behavior; however, despite countless interventions, the issue continues to be a problem today. Why then does the issue of disruptive behavior persist? We argue that one reason is the multiple ways disruptive behavior can be described, henceforth defined as the "plurality of terms", which can make it difficult to collect relevant data by doing a simple literature search. Hence, we believe having a single definition for “disruptive behavior” will improve the meta-analysis on disruptive behavior research.

## Introduction and background

Within the health care literature there exists countless articles and studies on the topic of disruptive behavior that strive to explain and describe the term and, in some cases, to propose solutions to the problem of these behaviors. However, despite the extensive research that exists on disruptive behavior and the corresponding efforts in combating this issue, the problem still persists within the clinical environment. We believe that there are many reasons for why this problem persists. One such reason is the plurality of terms that exist for disruptive behavior. We believe that such plurality limits a cogent meta-analysis of the research. Put another way, it is difficult to comprehensively research a problem in a conglomerate manner when that problem is described by a myriad of terms throughout the literature. Therefore, in this article, we strive to demonstrate that the creation of a single definition of disruptive behavior will allow for a more accurate and complete analysis of the problem by minimizing the plurality of terms that exist. This in turn will improve how we prevent, mitigate, and manage disruptive behavior. 

In this paper, we will first demonstrate that disruptive behaviors continue to occur within the health care environment. We will then outline the plurality of terms that are within existing medical literature that describe disruptive behaviors. Finally, we will illustrate that current disruptive behavior research is being negatively impacted by the varying definitions. But first it is important to provide a brief history of the term disruptive behavior.

## Review

The history of 'disruptive behavior' as a term

The exact term 'disruptive behavior' was used in literature as early as 1995 in the article “The Disruptive Physician: The Risk Manager’s Role” wherein it is stated that “… Examples of disruptive behavior are …” [1]. The term is again used in the 1999 article “The Disruptive Physician” when it states, in part, the “… Definitions of disruptive behavior vary, …” [2]. Subsequently, in June of 2000, the Council on Ethical and Judicial Affairs presented a report on "Physicians With Disruptive Behavior” to the Reference Committee on Amendments to Constitution and Bylaws within the American Medical Association (AMA) organization [3]. The report defined disruptive behavior, discussed intervention, and provided a conclusion with recommendations. However, simply because the term disruptive behavior was not first used until the mid-1990s does not mean that such behaviors did not occur prior to this time. For instance, on June 20, 1875, the The New York Times published an article entitled “Pugnacious Physicians” [4]. The article reported that following a session of the Academy of Medicine several doctors were charged with “… threatening personal violence to each other by assault with deadly weapons.” Another example is an article published on July 5, 1881 in The New York Times entitled “The Physicians Quarreling” [5]. This article details the actions of two doctors who became “… excited …” resulting in one doctor calling the other a liar and the other doctor jumping “… to his feet with hostile intent …”. This all occurred while President Garfield was in the adjoining room being treated for a gunshot wound. Lastly, in 1909 an article by The York Times entitled “The Hospital Tyrants and Their Victims, the Nurses” details the observations of two doctors on the negative behaviors of head nurses towards their student nurses [6]. Even though the article didn’t use the term disruptive behavior, there is no doubt that the actions described in the article were indeed disruptive. So, even as early as the 1800s, different terms were being used to describe disruptive behaviors. Before exploring the latter issue further, we must first demonstrate that disruptive behavior continues to be a problem within the health care environment today.

Existing research proves that disruptive behavior continues to occur

In 2002, Dr. Alan H. Rosenstein authored an article entitled "Nurse-Physician Relationships: Impact on Nurse Satisfaction and Retention" [7]. The article analyzed 1,200 responses from a survey of nurses, physicians, and hospital executives conducted by Voluntary Hospitals of America (VHA) West Coast. The survey was conducted to examine nurse-physician relationships with several questions in the survey focusing on disruptive physician behavior. Dr. Rosenstein determined that a surprising 92.5% of 1,177 respondents “... had witnessed disruptive behavior by a physician". The findings also showed that the most-cited instances of disruptive behavior witnessed or experienced by respondents (n = 1,200) were “... yelling or raising the voice, disrespect, condescension, berating colleagues, berating patients, and use of abusive language ..." Lastly, the respondents agreed that disruptive physician behavior “... influences nurses as well as other staff members’ attitudes toward patient care and inhibits teamwork, affecting the efficiency, accuracy, safety, and outcomes of care”.

Dr. Rosenstein revisits this topic in 2005 in a follow-up study designed to determine the impact of disruptive physicians and nurses [8]. The results show that 74% of 965 respondents (i.e., physicians, nurses, administrators) had witnessed disruptive behavior from a physician and 68% of 960 respondents indicated that they had witnessed disruptive behavior from a nurse. Furthermore, 60% of 1,487 respondents answered yes to the survey question: Are you aware of any potential adverse events that could have occurred from disruptive behavior? The survey results also showed that 17% of 1,441 respondents indicated that they were aware of a specific adverse event caused by disruptive behavior. Lastly, the authors state that “Most respondents perceived disruptive behavior as having negative or worsening effects, in both nurses and physicians, on stress, frustration, concentration, communication, collaboration, information transfer, and workplace relationships.”

Likely due to the increased awareness of this issue stemming from the aforementioned groundbreaking research, formal recognition of disruptive behavior by national regulatory organizations began in the late 2000s. For instance, in 2008 The Joint Commission issued a Sentinel Event Alert entitled "Behaviors that undermine a culture of safety" which stated that “Intimidating and disruptive behaviors in health care organizations are not rare.” [9]. Furthermore, the sentinel event stated that the “... presence of intimidating and disruptive behaviors in an organization, however, erodes professional behavior and creates an unhealthy or even hostile work environment – one that is readily recognized by patients and their families.” Also in 2008, the AMA included in its Model Medical Staff Code of Conduct the “… means for review and disciplining medical staff members for inappropriate or disruptive behavior.” [10]

Despite the aforementioned research and directives focused on disruptive clinicians since 2002, the problem persists. Dr. Rosenstein’s sentinel research of 2002 described above found that 92.5% of respondents “... had witnessed disruptive behavior by physicians.” [7]. Now, more than a decade later, current research shows that the disruptive behavior remains prevalent and continues to negatively impact patient care. Take, for example, a 2012 article co-authored by Dr. Rosenstein that describes survey results demonstrating that 57% of 370 respondents (i.e., Emergency Department (ED) physicians, nurses, other staff employees) had witnessed disruptive behavior by ED physicians and 52% had witnessed disruptive behavior by ED nurses [11]. Moreover, 13% of participants responded affirmatively to the question that they were “… aware of any specific adverse advent that occurred due to disruptive behavior.” Subsequently, a 2013 article reported that 73% of 885 respondents to a survey had witnessed their coworkers experiencing disruptive behavior and 84% of 1,559 respondents had experienced disruptive behavior during the past year [12]. It was also reported that 10.1% of 1,131 respondents indicated that harm to their patient had occurred due to disruptive behavior. The authors state that their findings “... add to the cumulative body of knowledge that this behavior undermines a culture of safety." Lastly the authors of the 2015 article entitled "Disruptive behavior within the workplace" report that 74% of 2,821 respondents (i.e., Licensed Practical Nurses (LPN), Registered Nurses (RN), Advanced Practice Registered Nurse (APRN)) had “… experienced some form of verbal abuse within the past 12 months …” with the majority of the perpetrators being “… either coworkers (45.6%) or managers (28.5%) …” [13].

This review provides strong evidence that disruptive behaviors continue to occur within the healthcare environment and negatively impacts patient care. Even though these articles provide considerable data showing that disruptive behaviors occur, we believe that they do not represent the full scope and magnitude of the problem. This is because the plurality of terms being used to describe disruptive behavior is limiting the meta-analysis of the research.

The plurality of terms for disruptive behavior: a review of existing literature

Earlier in this paper, we alluded to the fact that even as early as the 1800s, different words were being used to describe disruptive behavior. So, what exactly constitutes disruptive behavior and what are the plurality of terms, including those that are synonyms, which are being used for it throughout the literature? In a comprehensive review of the literature, we have found that the terms used to describe and define disruptive behavior are not only numerous but also vary quite drastically. We reviewed a total of 163 articles in which we discovered 207 unique terms that constitute disruptive behavior. The list of these disruptive behavior terms was created by (1) breaking down the respective authors’ definitions into their composite words and (2) including any examples of disruptive behavior that the authors explicitly provided (e.g. “Common types of disruptive behaviors include impatience with questions, …” [14]). Table 1 provides a small sample of these terms, which illustrates the wide spectrum of terms that exists (e.g., “racial slurs”, “refusing to perform tasks”, “voice intonation”, and so forth) to describe and define disruptive behavior in the literature.

**Table 1:** A sample of terms This table provides a sample of the 207 disruptive behavior terms that were collected from the 163 articles.

Disruptive Behavior Term	Total Count
racial slurs	2
disrespectful interaction	2
derogatory comments about organization	1
berating	3
degrading comments	3
publicly degrading team members	2
refusing to participate in facility programs	2
criticizing staff in public	4
persistent inappropriate behavior, rising to level of harassment	3
confrontation	2
idiosyncratic	3
refusing to perform tasks	5
inconsistent	2
occasional flirtation	1
humiliating	1
inappropriate behavior	4
aggression	2
facial expressions	2
negative comments about physician's care	3
noncompliance with existing policies	2
belittle	4
refusing to follow policies	4
voice intonation	3
impatience with questions	5
foul language	4
not able to work well with others	1
sexual comments/innuendo	3
harassment	3
bullying	4
nonverbal acts	4
frequently litigious	2
sarcastic	2
exhibiting uncooperative attitude	5

While Table 1 provides a small sample of the 207 terms, Table 2 shows the top 20 disruptive behavior terms and the corresponding totals for each occurrence in the literature. The term “sexual harassment” was citied 27 times and “intimidation” 24 times making them the first and second most citied terms in the literature. Also, reviewing Table 2 reveals that there are terms that can be associated within one another. For example, the terms “verbal abuse”, “yelling”, “cursing, swearing, profanity”, and “insulting comments” can be categorized as terms that are verbally abusive. Another example are the terms “physical threats”, “threatening violence/harm” and “threats”, all of which can be categorized under the common term threats. It is in such a manner that these 207 terms were categorized.

**Table 2:** Top 20 terms This table lists the 20 most citied disruptive behavior terms.

Disruptive Behavior Term	Total Count
sexual harassment	27
intimidation	24
throwing	16
physical abuse	14
physical threats	14
verbal abuse	13
condescension	12
yelling	11
physical behavior that negatively affects patient care	10
verbal behavior that negatively affects patient care	10
cursing, swearing, profanity	9
demeaning behavior	9
angry outbursts	8
failure to respond to phone calls	8
passive aggressive	8
threatening violence/harm	8
threats	8
abusive conduct	7
insulting comments	7
other harassment	7

Table 3 shows the eight categories that were created using the common themes from the 207 terms. It is important to mention that some terms were assigned to more than one category, which is why the total terms in Table 3 is greater than 207. Reviewing Table 3 reveals a category entitled "A pattern of passive aggressive behavior." This category’s name includes the word “pattern” because passive aggressive behavior requires a pattern to exist. For instance, the terms “ignoring behavior”, “ignoring pages/calls”, “failure to respond to pages”, and “failure to respond to phone calls” were all assigned to this category. Barring a pattern, these behaviors are not considered disruptive because there could be many benign, non-disruptive reasons for the behavior such as the pager battery being dead, someone having a busy or bad day, person is in the restroom, and so forth. To illustrate this point further, take the following true event as a contrasting comparison. On August 14, 2012, a physician took a piece of ice out of his mouth and placed it down the pants of a female employee [15]. This action does not require a pattern: it is disruptive on the first occurrence. In fact, it requires immediate organizational and perhaps law enforcement intervention. Subsequently, the physician’s license was suspended for at least six months due in part to this incident. This contrasts with the above examples given for passive aggressive behavior. In creating these eight categories, we have demonstrated that the plurality of terms that exist for disruptive behavior can be classified and organized. This is the first step in creating a single definition of disruptive behavior.

**Table 3:** The eight categories This table contains the eight categories, along with the total number of terms allocated, that were derived from the 207 disruptive behavior terms. Also, some terms were allocated to more than one category.

Category	Number of terms allocated
A pattern of passive aggressive behavior	129
Physical or verbal threats	25
Verbal abuse	25
Physical violence	18
Harassment	18
Intimidation	9
Bullying	8
Discrimination	8

We have thus far illustrated the plurality of terms that exist for the term disruptive behavior. We will now specifically examine the synonyms that exist for disruptive behavior within the literature. We again reviewed 163 articles and found five articles wherein the authors specifically state that a specific term was synonymous to the term disruptive behavior [16-20]. Within these five articles, eight terms were stated to be synonymous to disruptive behavior: lateral violence, incivility, bullying, horizontal violence, verbal abuse, hostility, nurse-to-nurse violence, and behaviors that undermine a culture of safety. For example, in the 2014 article entitled "Ending disruptive behavior: Staff nurse recommendations to nurse educators", it states “Other common synonyms for DB (Disruptive Behavior) include lateral violence, incivility, bullying, and horizontal violence” [16]. As such, the terms lateral violence, incivility, bullying, and horizontal violence were included in as terms that are synonyms of disruptive behavior. Another example can be found in the article "A Prescription for Disruptions in Care" wherein it states that “Disruptive behaviors can be displayed by any health care worker, and many terms, including bullying and horizontal violence, have been used to describe them” [20].

The existence of synonyms to disruptive behavior may result in missed literature items which thereby limits the meta-analysis of the topic. Furthermore, two of the synonymous terms, verbal abuse and bullying, are also categories depicted in Table 3. Having these two terms as both a category and a synonym of disruptive behavior provides another layer of confusion and negatively affects the analysis of disruptive behavior.

So far, we have illustrated that the terms that exist in the literature to describe and define disruptive behavior are numerous and vary greatly. Furthermore, it was shown that these terms can be succinctly organized into eight categories (Table 3). Finally, we have demonstrated that there exists eight synonyms of disruptive behavior within the literature. It is therefore fair to state that the plurality of terms and synonyms for disruptive behavior negatively impacts the analysis of disruptive behavior because they result in confusion on what constitutes disruptive behavior, the need for additional literature search categories, and disagreement on how the existing literature should be analyzed. Ultimately, this results in misdirection in the manner that future research on the topic is conducted. In order to illustrate the latter point, two specific examples of how the plurality of terms and synonyms of disruptive behavior have already negatively affected research on disruptive behavior are illustrated below.

Other existing research categories that should be classified as disruptive behavior research

Category - Nurse Verbal Abuse

Conducting a limited literature search on the term verbal abuse, which was deemed a category of disruptive behavior and a synonym as found in the literature, resulted in nine research articles [21-29]. After analyzing these nine research articles along with other supporting articles we were able to draw the conclusions (1) that there is a specific research category referred to as nurse verbal abuse and (2) that verbal abuse directed towards nurses from other clinicians and support staff does occur. For instance, an article published in 1987 entitled "Verbal Abuse in Nursing: Report of a Study" stated that of the 421 registered nurses surveyed, 48% were verbally abused by physicians [29]. Subsequently, in a 1991 article, 94% of 163 respondents indicated they had experienced verbal abuse from a physician “at least once” and “at least once every two or three months” [27]. Lastly, in a 1997 article entitled "Verbal Abuse of Staff Nurses by Physicians" the results showed that 90% of its 130 respondents experienced one episode of verbal abuse at least one time during the past year from physicians [23]. These studies provide empirical evidence that disruptive behavior occurred prior to 2002 because verbal abuse is a component of disruptive behavior as has been previously illustrated in this article. It is therefore both appropriate and necessary to include this and potentially other verbal abuse data in the analysis of disruptive behavior.

The above examples illustrate that research on and analysis of nurse verbal abuse is inherently a type of disruptive behavior research. Having a single definition of disruptive behavior would have automatically brought nurse verbal abuse research and related terms into the disruptive behavior research category. This would have eliminated missed literature searches on the topic, redundant research, and the wasteful use of resources. Most importantly, having a single definition would have provided a more complete analysis and discussion of disruptive behavior by automatically including all the relevant studies on the topic.

After analyzing the verbal abuse literature search, we decided it was necessary to conduct another parallel literature search. The purpose of this literature search was to determine if there were other research categories that complement disruptive behavior research that are not one of the eight synonyms of disruptive behavior. After a broad literature search, we determined that medical student mistreatment also referred to as medical student abuse was another research category that complements and, in fact, composes disruptive behavior research.

Category - Medical Student Mistreatment

**Figure 1:**
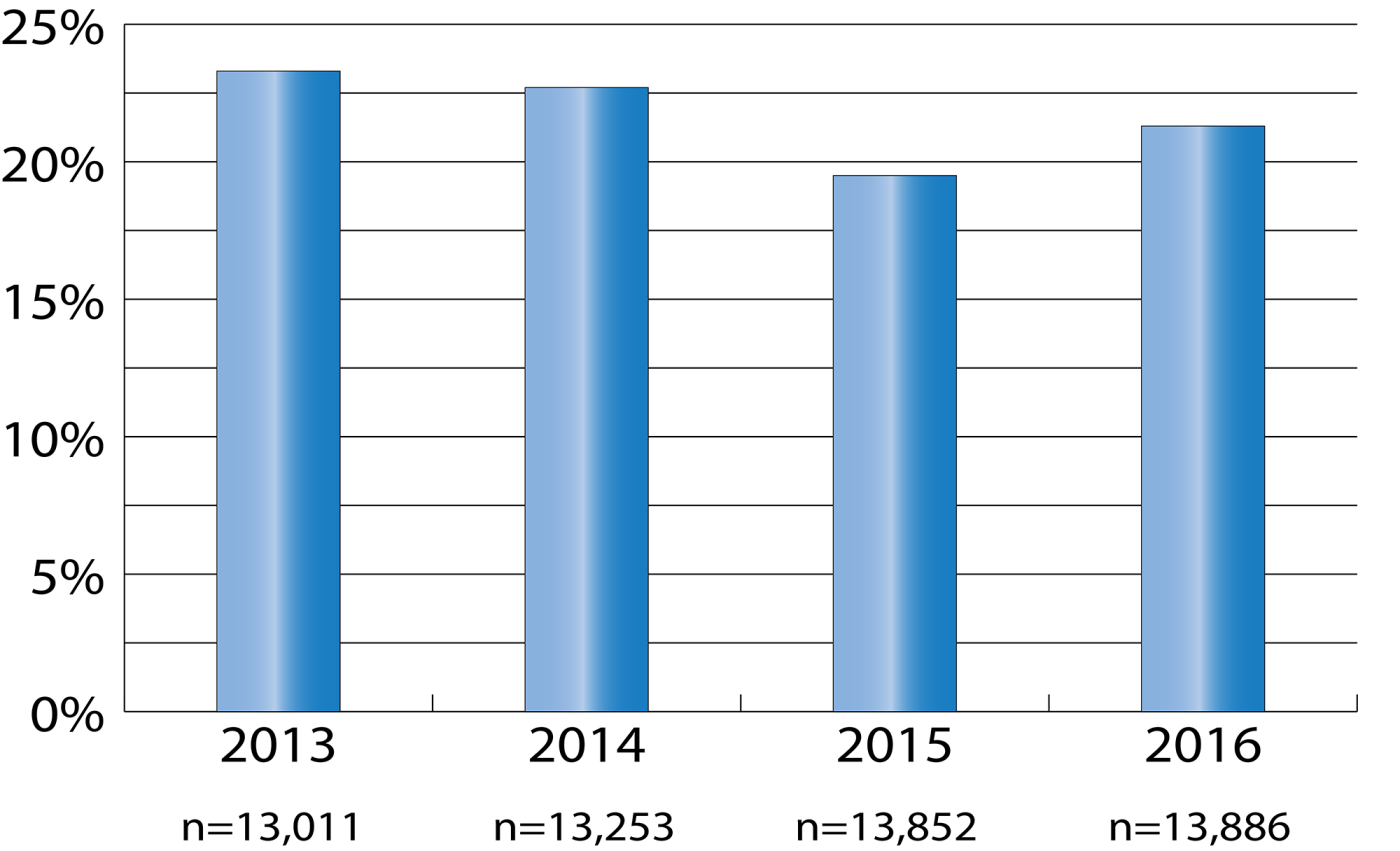
Students who were publicly humiliated The bar graph illustrates the percentage of students who reported in the Association of American Medical Colleges (AAMC) surveys for the years 2013 to 2016 that they were publicly humiliated once, occasionally, or frequently. [32-33, 36-37]

**Figure 2:**
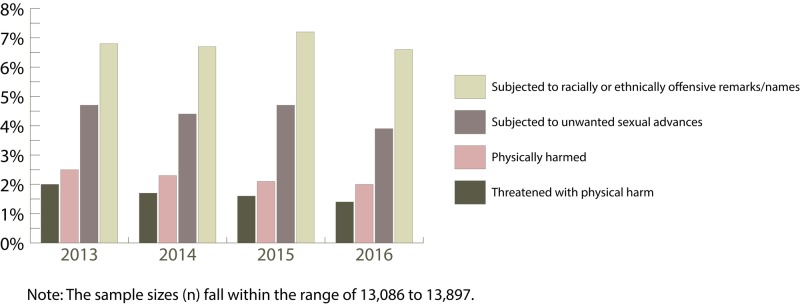
Experiences of students being mistreated The bar graph illustrates the percentage of students who reported in the Association of American Medical Colleges (AAMC) surveys for the years 2013 to 2016 that they were mistreated. [32-33, 36-37]

In 1982, a groundbreaking commentary was published in The Journal of the American Medical Association (JAMA) entitled “Medical Students and Medical School” [30]. The author posed a simple but important question, “Is it possible that medical school is a place where medical students are actually abused?” This statement, along within the entirety of the author’s commentary, was proactive at the time and became the catalyst for additional research into whether medical students are abused while in training [30, 31]. As such, our literature search resulted in the analysis of eight articles and four national surveys that demonstrate that medical students are indeed mistreated [31-43]. For example, the author of the 1982 commentary co-authored an article in 1984, which surveyed 50 medical students (preclinical and clinical) with respect to personal abuse they received from physicians [30, 31]. The results showed that all 25 clinical students “… had, to a variable degree, been personally abused and humiliated by residents or by faculty members” while one out of the 25 preclinical students had personally been abused [30, 31]. Subsequently, the results of a survey on the abuse that medical students received during their internal medicine clerkship were published in a 1999 article [39]. The authors stated that “… several female students objected to “inappropriate comments” made by male house staff.” A more horrific statement made by one female medical student was that she had experienced a resident “… feeling her leg under the table during a conference.” These experiences not only constitute types of disruptive behavior but may also constitute a criminal act. Lastly, since 1978 the Association of American Medical Colleges (AAMC) has been administrating a yearly survey to graduating medical students [44]. Starting in 1991, the survey began including questions on experiences of being mistreated by staff (faculty, nurses, residents/interns and other institutional employees) and other medical students [35]. Reviewing the survey data for the years 2013 to 2016 (See Figure 1 and Figure 2) illustrates that mistreatment (disruptive behavior) by staff and other medical students does occur during their training [32-33, 36-37]. Figure 1 shows that at least 19% of respondents for each year experienced being publicly humiliated, which is an example of disruptive behavior. Recall that the term humiliating was one of the 207 terms derived from our research in this paper (Table 1). Figure 2 depicts some distressing results from the survey. Specifically, it illustrates that some medical students experienced threats of physical harm due to disruptive behavior. Even though it was a small percentage of medical students who reported this behavior, threats of physical harm should never occur. Even more disturbing is that at least 2% of respondents for each year indicated that they had been physically harmed during their training, which is not acceptable.

The above discussion undeniably illustrates that medical students experience disruptive behavior during their training. Recall that searches for disruptive behavior did not pick up these studies. Rather, it was necessary to search using the term “medical student mistreatment” to find the studies that exist on this topic. Put another way, this very striking and rich category of disruptive behavior is easily missed in an analysis of the literature on disruptive behavior. Not utilizing medical student mistreatment research data very much limits the scope and depth of disruptive behavior research. As it currently exists, using two different research category names (medical student mistreatment and nurse verbal abuse) results in two parallel categories of research within the literature that should be included as one.

## Conclusions

In this paper, we have demonstrated that disruptive behavior has existed for a long time and continues to occur to the present time. The topic of disruptive behavior exists in the literature over that same time frame. These literary studies are searchable under a variety of terms that are beyond that of just “disruptive behavior”. Specifically, we have shown that there exists a plurality of terms used to describe and define disruptive behavior. This plurality of terms includes a subset of many synonyms of disruptive behavior. The existence of the multitude of these terms and synonyms has resulted in a limited understanding of disruptive behavior. We have therefore argued that having a single definition of disruptive behavior will improve the analysis and meta-analysis of the topic because it will ensure that all research that focuses on negative treatment of healthcare workers (clinicians, support staff, students) is searchable within the same research category and that future research is conducted within the same research category. Finally, we hope that this research will be the starting point in the development of a single definition.
